# Intravascular Large B-Cell Lymphoma Mimicking Temporal Arteritis

**DOI:** 10.1155/2018/5364985

**Published:** 2018-04-29

**Authors:** Ifeyinwa Emmanuela Obiorah, Metin Ozdemirli

**Affiliations:** Department of Pathology, Medstar Georgetown University Hospital, Washington, DC, USA

## Abstract

Intravascular lymphoma is a rare type of lymphoma, characterized by growth of lymphoma cells within the microvasculature. The majority of the cases are of B-cell lineage, although rare examples of T or NK lineage have also been reported. The lymphoma is usually widely disseminated in the vascular spaces of any organ at the time of diagnosis including the skin and bone marrow. Lymph nodes are typically spared. The clinical picture depends on the specific organ involvement making the correct diagnosis very difficult. Here, we report a case of intravascular large B-cell lymphoma diagnosed postmortem on a 69-year-old African-American male who presented with unilateral proptosis and visual loss. An initial diagnosis of temporal arteritis was made and the patient received corticosteroids. However, the patient developed multiorgan failure and expired. On autopsy, there was disseminated intravascular lymphoma involving predominantly vessels within the heart, kidneys, liver, stomach, lungs, adrenal glands, small intestine, bladder, thyroid, and brain. Interestingly, there was also partial involvement of the retroperitoneal lymph nodes which is an unusual presentation in this disorder. Immunohistochemical staining showed that the lymphoma cells were positive for CD20, indicating B-cell phenotype. This case supports the “mimicking nature” of this rare entity with an unusual presentation with proptosis and visual loss, simulating temporal arteritis and a rare involvement of the retroperitoneal lymph nodes. The presentation of intravascular large B-cell lymphoma can vary, and the key to diagnosis is dependent on histopathology and immunohistochemistry. Increased awareness, early tissue diagnosis, and prompt chemotherapy are crucial for this otherwise lethal disease.

## 1. Introduction

Intravascular large B-cell lymphoma (IVLBCL) is a rare type of non-Hodgkin's lymphoma that is characterized by selective growth of tumor cells in the lumen of small to medium vessels of various organs, the absence of lymphadenopathy, aggressive clinical behavior, delay of early and accurate diagnosis, and fatal consequences [1]. IVLBCL was first described in 1959 by Pfleger and Tappeiner [2], and it was considered to be endothelial in origin. In 1982, a lymphoid origin was suggested by Ansell et al. [3] due to their discovery of a surface membrane immunoglobulin on the tumor cells. However, the lymphoid nature of the disorder was confirmed by Wick et al. [4] in 1986, who demonstrated common leucocyte antigen on the surface of the malignant intravascular cells. The precise mechanism of the distinct presentation of IVLBCL still remains predominantly unknown. Thus, continued research is important to enhance better understanding of this malignant lymphoma. Due to the extremely rare nature of the disorder, reports of IVLBCL in the literature are usually made in the form of case reports and small case series. Based on a few large series, IVLBCL is classified into two main types: the Western type which frequently presents with neurologic and dermatologic features and the Asian variant which is characterized by hemophagocytic syndrome. Neurologic symptoms of IVLBCL, although more common in the Western variant of IVLBCL, can occur in 25% of the Asian type [5]. These symptoms are heterogeneous and include alteration of consciousness, motor and sensory deficits, seizure, paresis, dementia, intentional tremor, disorientation, and gating disturbance. Neurological symptoms are thought to be important features that can lead to an accurate diagnosis. Here, we present a very unusual type of IVLBCL with unilateral visual loss and proptosis, thus mimicking temporal arteritis. The lymphoma also involved the retroperitoneal lymph nodes which made the case very challenging because the disorder is known to present typically in extranodal sites. The disease entity needs to be considered in any patient presenting features such as visual loss and proptosis, especially in the presence of a biopsy-negative temporal arteritis.

## 2. Case Report

**Figure 1:**
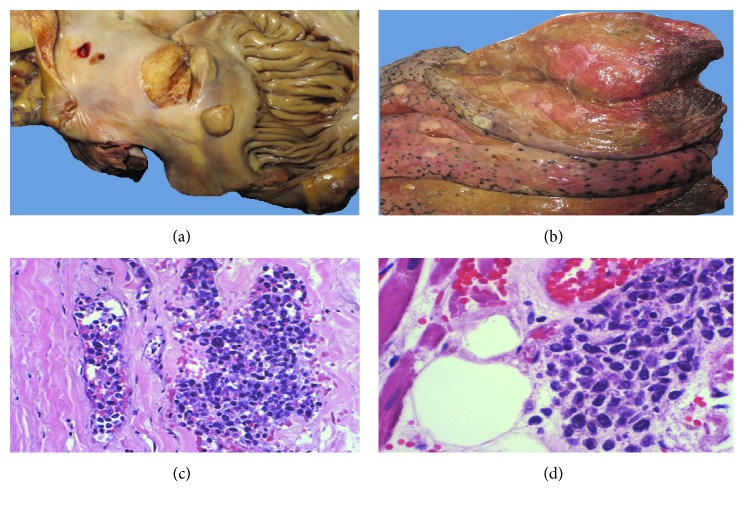
Gross and histologic examination of organs involved with lymphoma. (a) A pedunculated and a subendothelial lymphoma lesion observed in the right atrium. (b) Left upper lung with multiple tan-white tumor lesions. (c) The aorta, lymphoma cells in the small vessel (hematoxylin and eosin (H&E), ×100). (d) Left atrium vessel with neoplastic lymphoid infiltrate (H&E, ×200).

**Figure 2:**
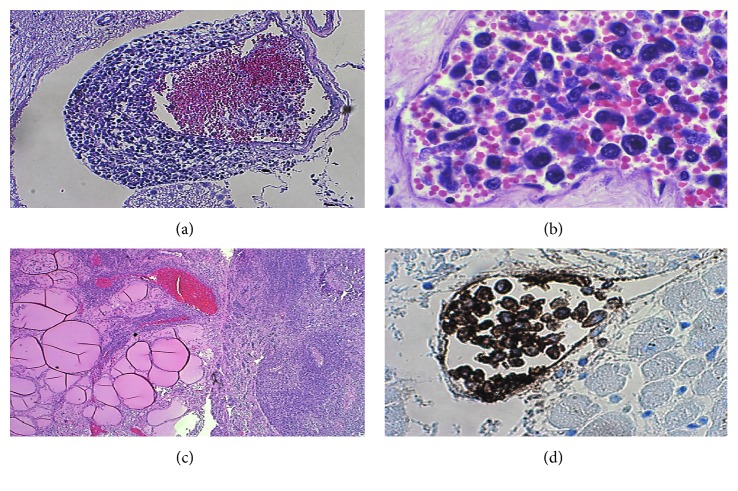
Histological and immunohistochemical staining of intravascular lymphoma cells. Small vessel lumen involved by lymphoma cells in the (a) brain (hematoxylin and eosin (H&E), ×100), (b) bladder (H&E, ×400), and (c) thyroid (H&E, ×40). (d) The neoplastic lymphoid cells in the coronary artery are positive for CD20 (H&E, ×200).

A 69-year-old African-American male presented to an outside institution with acute onset of right visual loss and mild right proptosis. His past medical history was significant for congestive heart failure, hypertension, asthma, arthritis, morbid obesity, and sleep apnea. Thyroid function tests were normal and a computed tomography (CT) scan did not reveal any retrobulbar mass. There was no evidence of stenosis or occlusion on magnetic resonance imaging (MRI). The patient was empirically treated with high-dose corticosteroid (60 mg prednisone daily) with visual improvement but subsequent temporal artery biopsy was negative for giant cell arteritis. The patient was discharged to home on oral prednisone 10 mg daily. Six weeks later, the patient was admitted to another outside facility due to congestive heart failure, gastrointestinal bleeding, and anemia. He had an emergency endoscopy with ablation and catheterization of a 1 cm ulcer. Shortly thereafter, the patient became hypertensive and developed encephalopathy and psychosis. Two weeks later, due to rapid deterioration of the patient's condition because of sepsis and multiorgan failure, the patient was transferred to our institution for higher level of care. On admission, the patient was febrile and the laboratory results obtained were as follows: white blood count 14.9 K/UL, platelet 140 K/UL, hemoglobin 8.7 g/dl, aspartate aminotransferase (AST) 68 U/L, alanine aminotransferase (ALT) 52 U/L, alkaline phosphatase 108 U/L, blood urea nitrogen 104 mg/dl, and creatinine 3 mg/dl. Chest X-ray showed bilateral pulmonary infiltrates; CT scan of thorax, abdomen, and brain showed only pulmonary infiltrates and was relatively unremarkable apart from a few scattered retroperitoneal lymph nodes. Due to the poor status of the patient, the lymph nodes were not biopsied at the time. During his hospital course, the patient was empirically treated for giant cell arteritis, sepsis secondary to pneumonia, anemia, acute and chronic renal failure, and thrombocytopenia. Despite continuous intensive treatment, the patient developed severe septic shock, multiorgan failure, and metabolic acidosis and expired two weeks later. On autopsy, subendothelial and pedunculated lesions were seen in the right atrium of the heart (Figure 1(a)). An organizing pulmonary thromboembolus was found in the apical vascular segment of the left upper lobe with a surrounding wedge area of infarct. Multiple tan-to-pale white tumor lesions were seen in the left lung (Figure 1(b)), right lung, right and left kidneys, retroperitoneal lymph nodes, right liver lobe, stomach, right and left adrenal glands, small intestine, bladder, thyroid, and brain. Histologic sections of the lesions showed disseminated neoplastic lymphoid cells in the lumen of small and intermediate vessels in the organs including the aorta (Figure 1(c)), right atrium (Figure 1(d)), brain (Figure 2(a)), bladder (Figure 2(b)), thyroid (Figure 2(c)), right and left kidneys, retroperitoneal lymph nodes, right liver lobe, stomach, right and left lungs, right and left adrenal glands, and small intestine. There was focal parenchymal involvement of the organs. The atypical lymphocytes were positive for CD20 (Figure 2(d)) on immunohistochemical staining and negative for CD3, CD5, CD10, CD56, MUM-1, and cytokeratin. Based on these findings, the final diagnosis of the patient's cause of death was multiple organ failure due to occlusion of the vessels by disseminated intravascular large B-cell lymphoma.

## 3. Discussion

Intravascular large B-cell lymphoma (IVLBCL) is commonly a fatal disease characterized by aggressive course and short outcome. It is a rare type of non-Hodgkin's lymphoma (NHL). The WHO classification of hematopoietic tumors defines IVLBCL as an extranodal diffuse lymphoma characterized by the presence of neoplastic lymphocytes only in the lumen of small vessels, particularly capillaries [1]. The clinical manifestations of this systemic lymphoma are extremely variable and the clinical presentations are related to the preferentially involved organs with various systemic symptoms, such as fever of unknown origin, general fatigue, marked deterioration in performance status, and neurological alteration [6]. Typically lymph nodes are not involved by IVLBCL but only a few cases have been reported [7]. The atypical presentation of the disorder often delays clinical recognition and due to its aggressive behavior, the diagnosis is made postmortem in half of the cases. IVLBCL is considered a disseminated disease at diagnosis, and it has a tendency to involve CNS and cutaneous symptoms in Western countries or present with hemophagocytic syndrome, bone marrow involvement, fever, hepatosplenomegaly, and thrombocytopenia in Asian countries, mainly in Japan. The variable clinical presentation in this disease is attributed to the geographical origin of the patients which makes the entity very intriguing. Hemophagocytic syndrome is extremely rare in Western countries and is a common feature in the Asian variant. This has been associated with higher production of inflammatory cytokines, including interferon-*γ*, tumor necrosis factor-*α*, interleukin-1*β*, and soluble interleukin-2 receptor (sIL2R) in Asian patients [8], thus leading to a systemic inflammatory response. These two different patterns of presentation do not correlate with patient survival with the exception of the cutaneous variant, which displays a better prognosis [9]. In our report, although our patient presented with neurological symptoms, it was an unusual form of presentation. Unilateral visual loss and proptosis are rare forms of presentation. Mills et al. [10] reported the only other known case of IVLBCL that presented with unilateral visual loss in an 88-year-old female. However, unlike our case, the patient had a left intraorbital mass and hence did not mimick a temporal arteritis. In addition, our patient had anemia, thrombocytopenia, and fever. These features are more common in the Asian variant. Here, it appears that there may be an overlap of the two variants of IVLBCL in our patient. However, these clinical and laboratory findings in IVLBCL are usually nonspecific and can be found in both variants of IVLBCL.

The key to diagnosis of IVLBCL is tissue biopsy. Since the lymphoma can affect any organ, timely selection of an organ for diagnosis by the clinician is imperative for early diagnosis. Similar to most reported cases, CT scan and MRI were not particularly helpful in our patient. In the Asian series, the most relevant diagnostic site appears to be the bone marrow [7]; however, random skin biopsies have proved to be a useful diagnostic tool in both the Western and Asian cohorts [11, 12]. Most cases of IVLBCL express B-cell-associated antigens such as CD19, CD20, and CD79a. Rare cases have reported intravascular T-cell or natural killer lymphoma [13, 14], but these have been thought to represent a disease entity different from IVLBCL. In a cohort of 96 patients with IVLBCL [7], almost all cases were positive for CD20, and CD5, CD10, Bcl-6, MUM-1, and Bcl-2 were positive in 38%, 13%, 26%, 95%, and 91% of the tumors, respectively. Similar to cases of diffuse large B-cell lymphoma, almost all the CD10-negative cases were MUM-1 positive, and they were considered to be of the nongerminal type. Cases of IVLBCL with CD5 positivity were associated with a higher frequency of marrow/blood involvement and thrombocytopenia and a lower frequency of neurologic abnormalities among patients with CD10-negative cases [7]. However, our patient was CD5 and CD10 negative and presented with predominant neurologic features of an unusual presentation. Patients with IVLBCL usually present with a disseminated disease, and treatment with a combination of chemotherapy (either anthracycline-based chemotherapy or cyclophosphamide, vincristine, doxorubicin, and prednisone combination chemotherapy) with rituximab has shown favorable outcome. In a clinical study of 57 patients receiving chemotherapy without rituximab and 49 patients receiving chemotherapy with rituximab, the progression-free survival (PFS) and overall survival at 2 years in patients receiving chemotherapy with rituximab were 56% and 66%, respectively. In the patients who received chemotherapy without rituximab, the PFS and overall survival at 2 years were 27% and 46%, respectively. In another study, Ferreri et al. reported that the overall survival at 3 years was 81% in 33 patients who received anthracycline-based chemotherapy and rituximab [15]. Improved survival is associated with early recognition and diagnosis of the intravascular lymphoma. We report a challenging case of IVLBCL with unilateral proptosis and visual loss, which was clinically diagnosed as temporal arteritis. IVLBCL should be considered in the differential diagnosis of any case of presumed (biopsy-negative) temporal arteritis. The most reliable criteria for diagnosis are morphology and immunohistochemical analysis on tissue biopsy which can demonstrate clusters of atypical large lymphocytes in vessels that often stain for B-cell markers as this case illustrates. Therefore, it is crucial to have an early tissue diagnosis and prompt chemotherapy to increase the survival rate from this rare and deadly entity.
